# Programmed Self-Assembly of DNA Nanosheets with Discrete Single-Molecule Thickness and Interfacial Mechanics: Design, Simulation, and Characterization

**DOI:** 10.3390/molecules28093686

**Published:** 2023-04-24

**Authors:** Keitel Cervantes-Salguero, Yair Augusto Gutiérrez Fosado, William Megone, Julien E. Gautrot, Matteo Palma

**Affiliations:** 1 Department of Chemistry, School of Physical and Chemical Sciences, Queen Mary University of London, London E1 4NS, UK; 2 School of Physics and Astronomy, University of Edinburgh, Peter Guthrie Tait Road, Edinburgh EH9 3FD, UK; 3 School of Engineering and Materials Science, Queen Mary University of London, Mile End Road, London E1 4NS, UK

**Keywords:** DNA nanotechnology, self-assembly, nanosheets, molecular dynamics, atomic force microscopy, fluorescence microscopy, interfacial shear rheometry

## Abstract

DNA is programmed to hierarchically self-assemble into superstructures spanning from nanometer to micrometer scales. Here, we demonstrate DNA nanosheets assembled out of a rationally designed flexible DNA unit (F-unit), whose shape resembles a Feynman diagram. F-units were designed to self-assemble in two dimensions and to display a high DNA density of hydrophobic moieties. oxDNA simulations confirmed the planarity of the F-unit. DNA nanosheets with a thickness of a single DNA duplex layer and with large coverage (at least 30 μm × 30 μm) were assembled from the liquid phase at the solid/liquid interface, as unambiguously evidenced by atomic force microscopy imaging. Interestingly, single-layer nanodiscs formed in solution at low DNA concentrations. DNA nanosheet superstructures were further assembled at liquid/liquid interfaces, as demonstrated by the fluorescence of a double-stranded DNA intercalator. Moreover, the interfacial mechanical properties of the nanosheet superstructures were measured as a response to temperature changes, demonstrating the control of interfacial shear mechanics based on DNA nanostructure engineering. The rational design of the F-unit, along with the presented results, provide an avenue toward the controlled assembly of reconfigurable/responsive nanosheets and membranes at liquid/liquid interfaces, to be potentially used in the characterization of biomechanical processes and materials transport.

## 1. Introduction

Nanosheets at solid and liquid surfaces can be utilized in biotechnology as scaffolds to promote cell cultivation and manufacturing [1]; for investigating biomechanical processes; and in materials science as membranes for freshwater production [2], doped films [3], and conducting electrodes [4]. An ideal nanosheet requires good adhesion to a surface and an extensive surface area coverage. Engineering nanosheets placed at dynamic liquid surfaces and with programmable mechanical responses requires nanosheets with sensitivity to mechanical stimuli, which in turn can be achieved by reducing the thickness of the nanosheet down to individual nanoparticles or molecules. Additionally, responsive nanosheets require the rational design of a basic unit that can be (bio)functionalized and is repetitive throughout the nanosheet. These requirements of an ideal nanosheet can be successfully achieved by harnessing the self-assembly of molecular units.

Molecular self-assembly is a powerful bottom-up process to fabricate nanosheets composed of diverse classes of molecular units that form according to physicochemical interactions. As examples of units, amphiphilic and DNA molecules have been used. Amphiphilic molecules are commonly used to fabricate membranes, which can further be integrated with top-down lithography fabrication to achieve desired morphologies [5]. DNA is used as a programmable material to fabricate precise and functional structural units by harnessing the programmability of DNA base sequences [3,6,7,8,9,10,11,12,13,14,15,16,17,18,19,20,21,22,23,24,25,26,27,28,29,30,31,32]. By purely using DNA, two-dimensional (2D) DNA nanosheets [16,17] and finite-size sheet-like nanorings [10], with the thickness of a single DNA duplex and connected on surfaces via DNA blunt-end stacking interactions, have been devised. Moreover, DNA-amphiphile composite nanosheets and nanoribbons with a thickness larger than that of a DNA duplex were self-assembled in the liquid phase via intercalation [19] and along one dimension at the air/aqueous interface via nonspecific interactions [11], respectively. In addition, the features of resulting DNA nanostructures and lipid surfaces can alter the adhesion strength promoted by electrostatic interactions [18].

In DNA nanotechnology, DNA self-assembly can be further advanced by the programmability of sticky-end cohesion to allow selective binding and permanent connectivity between DNA units to form nanosheets in a hierarchical and programmable way [3,16,20,21]. Small DNA strands protruding from each unit, called sticky ends, allow the selective binding of units via DNA base pairing. Whereas using large and relatively robust DNA units, such as DNA origami, provide abundant space per unit for molecular arrangement and functionality [8,9,12,13,14,15,16,21,22], small units provide simplicity and a large quantity of units per volume as they are composed of a few addressable DNA strands [3,20,23,24,25,26,27]. The addressability of such strands may provide a high density of functional moieties per unit in a large nanosheet, which can help with promoting adhesion to surfaces. Small units can be brick-like [3,20] or branched structures [24]. Branched structures have multiple arms connected through a junction. Among the different examples of branched units, X- and Y-shaped units are of interest as their topology may promote nanosheets at low DNA concentrations, in contrast to the well-known gelation at elevated concentrations [28,29]. X-shaped units, consisting of four DNA duplex arms, can form nanosheets with “rhombic area” porosity and reconfigurability [25]. To the best of our knowledge, nanosheets assembled out of Y-shaped units consisting of three DNA duplex arms in a junction and connected via sicky ends have not been reported, presumably due to the flexibility in the junction. Instead, large-area coverage with “hexagonal area” porosity via solid-surface-mediated self-assembly of rigid Y-shaped units was reported [26]. To produce nanosheets with large surface coverage and adhesion on varied surfaces, the hierarchical and programmed self-assembly of simple DNA units with functional moieties needs to be investigated.

In turn, the assembly of macroscopic objects based on the rational design of DNA assemblies can enable the control of mechanical properties, and potentially of responsiveness, with nanoscale resolution. Recently, the control of interfacial mechanics has been proposed to enable the emergence of new technologies. For example, the structuring of liquids via interfacial jamming of nanoparticles has enabled the design of liquid/liquid microfluidic platforms [33,34]. Another example is the regulation of interfacial shear strength, elasticity, and toughness to enable the culture of adherent cells at the surface of liquids [35,36,37]. Indeed, the generation of protein nanosheets displaying sufficiently strong and elastic mechanical properties, in addition to bioactivity, in order to resist the mechanical shear forces generated by cells during spreading and proliferation has enabled the long-term culture of stem cells and the maintenance of their phenotype [38,39]. Although some of the rules enabling the control of interfacial mechanics and elasticity are starting to emerge, the control and prediction of interfacial mechanics remain difficult. Platforms that enable the rational design of the mechanics of liquid/liquid interfaces may therefore have useful applications for microfabrication and biotechnologies.

**Scheme 1:**
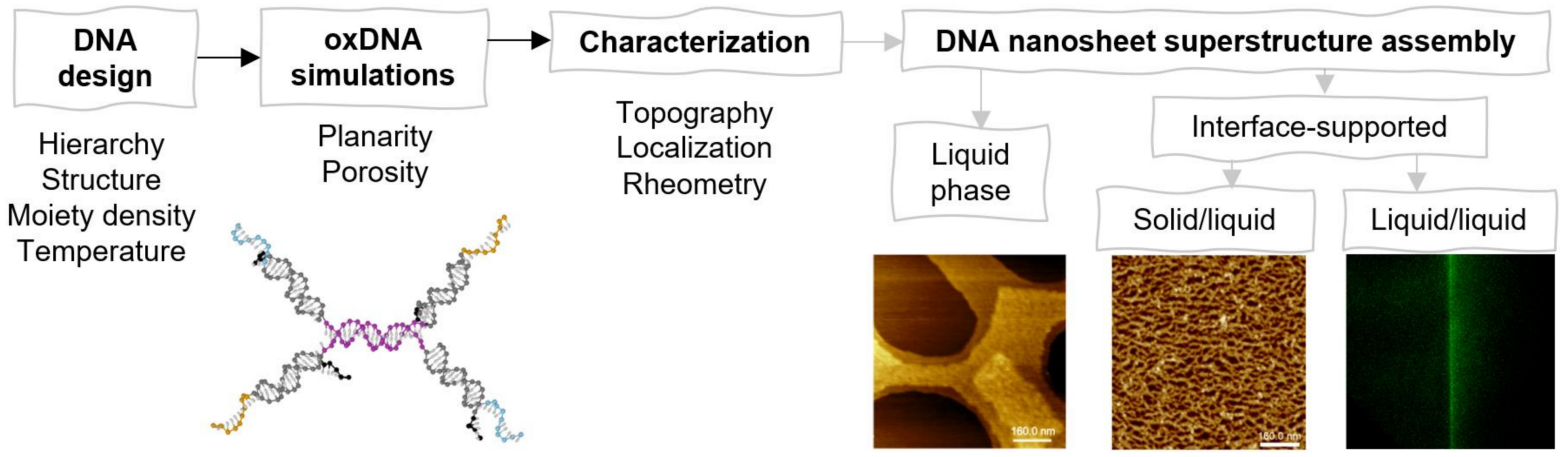
Workflow indicating key aspects of the present study: design, simulation, and characterization of the DNA nanosheet superstructures assembled at different environments.

Here, we designed, simulated, and characterized DNA nanosheets formed in different environments (see Scheme 1). By following a hierarchical self-assembling approach, we fabricated DNA nanosheets that are self-assembled out of DNA units, which are minimally composed of three strands. The unit is called an F-unit, owing to its resemblance to a Feynman diagram as well as denoting flexibility. The F-unit was designed to maximize the density of molecular functionalization to enable assembly at liquid/liquid interfaces while at the same time readily enabling modifications. By using molecular dynamics (MD) simulations, we examined crucial aspects of the F-unit structure, such as planarity and porosity. Experimentally, nanosheets were assembled in the liquid phase, at the solid/liquid interface, and at the liquid/liquid interface. Each step in the topographical characterization of the nanosheets enabled us to tune the conditions for optimizing nanosheet formation. Successful formation was achieved at the liquid/liquid interface, and its interfacial mechanical properties were characterized via interfacial shear rheology.

## 2. Results and Discussion

### 2.1. DNA Design

**Figure 1:**
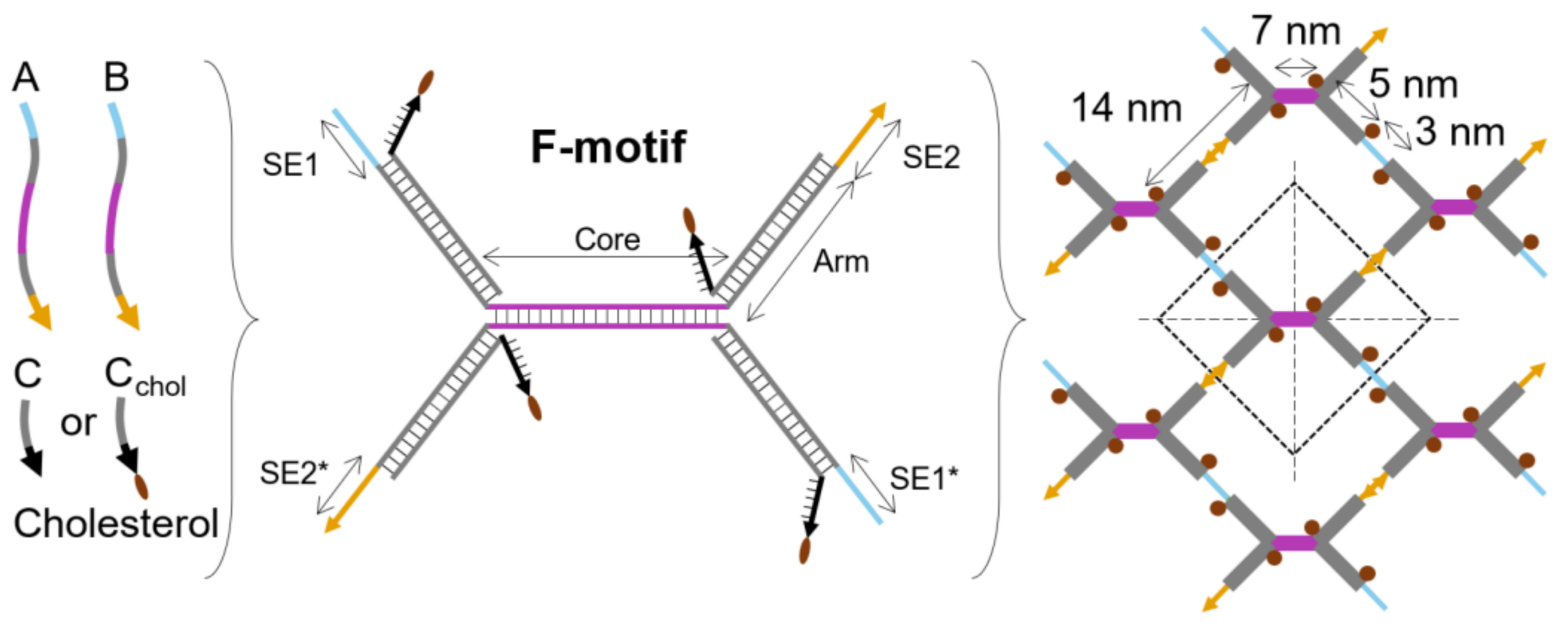
Hierarchical and programmed self-assembly of DNA nanosheets. Three DNA strands A, B, and C self-assemble into an F-unit. F-units further self-assemble into larger structures. The F-unit has one core, four arms, and four sticky ends (SE1, SE1*, SE2, and SE2*).

In our hierarchical approach to fabricate DNA nanosheets, we programmed the sequences of three unique DNA strands to form an F-unit (Figure 1). The three DNA strands are strands A, B, and C (Figure 1). From a functional perspective, the F-unit consists of three parts: core, arms, and sticky ends (SEs). A single F-unit has one core, four arms, and four sticky ends. The core and the arms are DNA duplexes. Strands A and B are partially complementary and form the core (purple DNA duplex). The arms are complementary to strand C (gray DNA duplex). The unpaired sequences of the strands A and B work as the sticky ends. Two sticky ends are complementary to each other (SE1 and its complementary SE1* in blue) as well as the remaining two sticky ends (SE2 and its complementary SE2* in yellow). The duplex lengths of the parts were selected in such a way that, after heating up the mixture of three DNA strands and cooling down, the core hybridizes first, then the arms, and finally the sticky ends. The structural periodicity of a DNA duplex was considered in the design, as this is important to assemble larger structures [40]. The core consists of 21 base pairs (bps), the arms have 16 bps, and the sticky ends have 10 bps (see Section 3, Text S1 for DNA sequences, and Table S1 for melting temperatures).

Structurally speaking, the F-unit can be considered as a double Y-shaped unit; however, half of the F-unit is different than a Y-shaped unit [19,29], as one of the strands along two arms is split. This feature enables the chemical functionalization of the DNA-strand components of the F-unit. A hydrophobic molecule can be covalently attached to each of the two split strands at their 3′ end. Considering that the F-unit forms a planar structure, our design was selected to minimize the distance between hydrophobic moieties used for attaching the nanosheet to a liquid surface, i.e., our design enables the nanosheet to be placed at the liquid/liquid interface. In our design, the minimum distance is about 3 nm, and the maximum distance is 14 nm. Shorter distances are possible by using special chemical modifications, but we opted to use commercially available DNA modifications. In the next section, to substantiate our rational design of the F-unit, we investigated the structural aspects of the designed single-layer DNA nanosheet using oxDNA simulations.

### 2.2. Structure and Planarity of Single-Layer DNA Nanosheets Validated by oxDNA Simulations

Whereas single-molecule characterization techniques are often used to resolve the structure of individual DNA molecules and complex DNA nanostructures, MD simulations can additionally provide insights into the structure, flexibility, and dynamics of a system [8,41,42,43]. This makes MD simulations a useful computational technique for assisting and evaluating the design of DNA nanostructures. Particularly, MD in the framework of the oxDNA coarse-grained model allows the monitoring of the configurations of single DNA constructs and the structure of large assemblies [41,42,43].

#### 2.2.1. Single F-Unit

**Figure 2:**
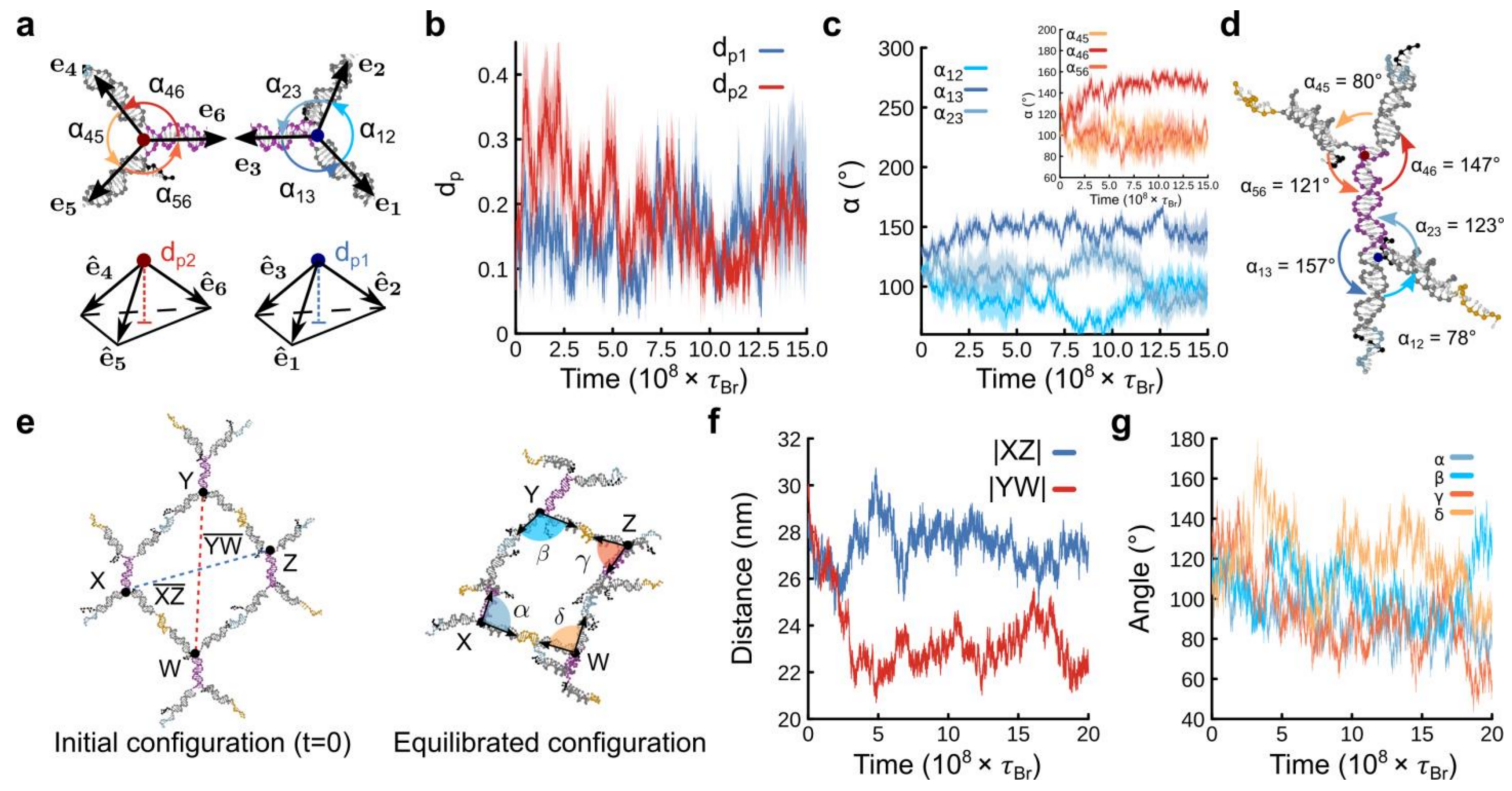
oxDNA simulations of a single F-unit (top panels (**a**–**d**)) and single pore (bottom panels (**e**–**g**)) of the nanosheet. (**a**) Top panel shows a schematic representation of the vectors **e_1_**, **e_2_**, and **e_3_** pointing from the junction to the last base pair in each arm. The angles *α_ij_* (with *j* > *i* and *i*, *j* = 1, 2, 3) between pairs of arms are also shown. Analogous vectors (**e_4_**, **e_5_**, and **e_6_**) and angles are defined for the other half of the molecule. Bottom panel represents the distance *d_p_*_1_ (or *d_p_*_2_) from the junction to the plane touching the end of the normalized vectors ${\hat{e}}_{1}$, ${\hat{e}}_{2}$, and ${\hat{e}}_{3}$ (or ${\hat{e}}_{4}$, ${\hat{e}}_{5}$, and ${\hat{e}}_{6}$). We note that the vectors are normalized *d_p_*_1_ ∈ [0, 1]. The distance *d_p_*_2_ is similarly defined for the top part of the molecule. (**b**) Time evolution of *d_p_*_1_ and *d_p_*_2_. (**c**) Main figure shows the temporal evolution of angles between arms *α_12_*, *α_13_*, and *α_23_*. Inset shows results for *α_45_*, *α_46_*, and *α_56_*. (**d**) Snapshot from simulations (at t = 1.5 × 10^8^*τ_Br_*) representing an equilibrated configuration of an F-unit. The shaded area enveloping the curves in panels (**b**,**c**) is the standard error computed over five different replicas. (**e**) Snapshots from simulations close to the beginning and after equilibration (t = 1.5 × 10^8^*τ_Br_*). The nanopore of the nanosheet was represented by four junctions (X, Y, Z, and W). (**f**) Time evolution of the magnitude of the segments $\bar{\mathrm{XZ}}$ and $\bar{\mathrm{YW}}$. (**g**) Time evolution of the angles *α*, *β*, *γ*, and *δ* sketched in panel (**e**).

Planarity and arms’ orientations are two geometrical features that characterize the configuration of a single F-unit (see Figure 2). To evaluate planarity, we monitored two distances, *d_p_*_1_ and *d_p_*_2_, each corresponding to a junction of the F-unit (see Figure 2a); a similar methodology was used in [43] (the timescale of the trajectories in Figure 2 is expressed in Brownian time (*τ_B_*), which is the time that a simulated sphere takes to diffuse a distance equal to its diameter). Each of these distances is defined as the magnitude of the vector pointing from the junction to the plane touching the tip of the three unitary vectors representing the direction of the three DNA arms closer to the same junction (see Figure 2a). When *d_p_* = 0, the three arms lie in the same plane, indicating a perfectly planar structure. As *d_p_* increases, the arms form a nonplanar pyramid-like structure. The temporal evolution of *d_p_*_1_ and *d_p_*_2_ is depicted in Figure 2b. We observed that at late times in our simulations, *d_p_*_1_ and *d_p_*_2_ reached an equilibrium value close to 0.2, indicating that the molecule exhibited very small out-of-plane fluctuations.

To determine the arms’ orientations, the angles between consecutive pairs of DNA duplexes (*α_ij_* with *j* > *i* and *i*, *j* = 1, 2, 3) were calculated over time (Figure 2c). At long times after equilibration, *α_13_* and *α_46_* are close to 150°, i.e., the simulations converged to a configuration in which the DNA arms with the cholesterol terminal located far from each junction are approximately aligned with the core of the F-unit (depicted with a purple backbone Figure 2a). *α_23_* and *α_56_* are close to 120°, i.e., the arms with a cholesterol terminal located close to the junction (represented by **e_2_** and **e_5_**) do not align with the core of the F-unit. The rest of angles, *α_12_* and *α_45_*, show values close to 80°. By summing all the angle values around each junction, we obtained 350°, which is close to 360°, supporting our findings that each junction is close to a planar structure. Figure 2d shows a representative configuration at later simulation times.

#### 2.2.2. Single Nanopore of Nanosheet

We might expect that when several F-units are assembled into a larger structure, each of these F-units would not necessarily display the equilibrium configuration we described in the previous section. Therefore, we investigated a larger structure that constitutes a nanopore of the nanosheet, formed by the assembly of four F-units. Figure 2e shows two representative configurations close to the beginning and end of the simulations. Moreover, examining the time evolution of the molecular components enabled us to extract the structural features including pore geometry and planarity.

To characterize the pore geometry, the nanopore was represented by four junctions (X, Y, Z, and W) belonging to four different F-units and defining a quadrilateral. The size of the nanopore was approximated by the diagonal lengths of the quadrilateral, $\bar{\mathrm{XZ}}$ and $\bar{\mathrm{YW}}$, which were 27.5 ± 0.9 nm and 23.0 ± 1.5 nm after equilibration, respectively (Figure 2f). The geometry of the nanopore could be monitored by the time evolution of the angles *α*, *β*, *γ*, and *δ*, which are angles between the pairs of arms internal to the pore (Figure 2g). At late times, three of these angles (*α*, *β*, *γ*) fluctuated around 90° ± 20°, whereas the other one *δ* was 130° ± 10°. This difference arose from the $\bar{\mathrm{XY}}$ side being not straight.

Determining the pore planarity required examining the planarity of the quadrilateral. If the arms connecting the junctions are collinear and lying in the same plane due to the quadrilateral geometry of the pore, then we would expect *α* = *γ* and *β* = *δ*. As shown above, only *δ* was different. Therefore, to support this finding, we applied an additional method in which we calculated the plane that passed through each corner of the quadrilateral and then determined how parallel those planes were. To achieve this, we calculated the unitary normal vectors associated with the plane passing through each corner of the quadrilateral and then computed the angles between the combinations of pairs of unitary normal vectors. A perfectly planar quadrilateral would yield angles of 0°. We found that the angles were close to 20° at late times, which strongly suggested the planarity of the quadrilateral, i.e., the planarity of the nanopore.

By considering all the results, our simulations strongly suggested that, at late times, the quadrilateral geometry of the nanopore is close to a parallelogram whose sides are slightly deviated from straight lines (see equilibrated configuration in Figure 2e). This is probably because the contour length distance between points X and Y (or Z and W) is 63 base pairs, roughly half of the persistence length of DNA, which may cause the deviations from rigid double-stranded DNA arms in these segments of the pore. We should expect similar planarity for a larger superstructure due to the hierarchical self-assembly of repetitive F-units. It is worth noting here that an interaction with a solid surface should minimize the fluctuations in the whole structure and promote a planar configuration, as we will see in the next section.

### 2.3. Self-Assembly in the Liquid Phase

We employed two strategies for self-assembling the nanosheets (Figure 1): self-assembly in the liquid phase and interface-supported self-assembly. The latter included solid/liquid and liquid/liquid interface-supported self-assemblies. The samples were prepared by mixing and annealing the three DNA strands at different stoichiometric ratios. We denote the different samples as ABC(m:n:p), where the ratio of the concentrations of strands [A]:[B]:[C] were m:n:p, where m = n = p/10. The ratio [A]:[B] was chosen as 1:1 and [A]:[C] as 1:10. This was due to the F-unit design, as A and B strands are structurally the same; therefore, there was no need to examine the case in which [A] is different than [B]. In each F-unit, the number of C strands is four times the number of A strands (or B strands); in our work, [C] = 10 × [A] (or 10 × [B]) was selected to have an excess of strands to ensure proper formation of the F-unit. The samples were annealed using either fast annealing protocol A, B, or C (FAP-A, FAP-B, or FAP-C) or slow annealing protocol A or B (SAP-A, SAP-B) (see Section 3). Atomic force microscopy (AFM) was used to characterize the DNA superstructures assembled in solution and at the solid/liquid interface (see Section 3).

**Figure 3:**
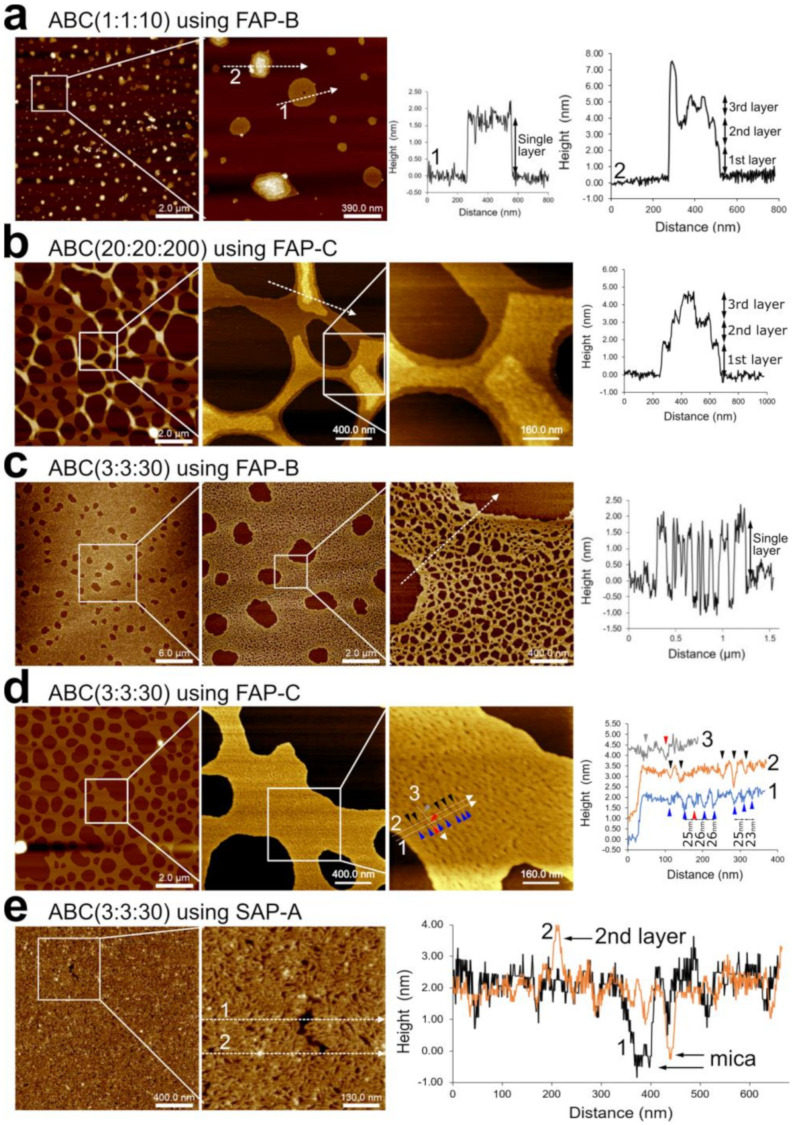
AFM characterization of DNA nanosheets self-assembled in the liquid phase. (**a**) ABC(1:1:10) with FAP-B showing single layer nanodiscs and multilayer particles with representative height profiles labeled as 1 and 2. [A] = 50 nM. (**b**–**d**) Zoomed-out scans show nanosheets with complete surface area coverage. (**b**) ABC(20:20:200) with FAP-C showing multilayer nanosheets. [A] = 1 µM. A representative height profile emphasizing three layers of the nanosheet is shown. (**c**) ABC(3:3:30) with FAP-B. Zoomed-in scan shows porous nanosheet. A representative height profile is shown. (**d**) ABC(3:3:30) with FAP-C. Zoomed-in scan shows compact nanosheet. Three representative height profiles labeled as 1, 2, and 3 are shown. (**e**) ABC(3:3:30) with SAP-A. Zoomed-in scan shows a large defect. Representative height profiles labeled as 1 and 2 are shown. In ABC(3:3:30), [A] = 150 nM.

The superstructures assembled in the liquid phase were characterized first. At a low concentration, sample ABC(1:1:10) using FAP-B showed disc-shaped superstructures lying flat on mica (Figure 3a). Interestingly, the discs had a thickness equivalent to the diameter of a single DNA duplex (height profile 1, Figure 3a). Some of the structures had asymmetric shapes whose thickness was resolved to multiple discrete DNA duplex layers (height profile 2, Figure 3a). Using FAP-C led to similar discrete multilayered structures and tubular structures (Figure S1a). Using the slower annealing protocol SAP-B for ABC(1:1:10), we observed spherical nanoparticles ~3 nm in height and tubular structures as well (Figure S1b). By decreasing the concentration as in ABC(0.6:0.6:6) and using SAP-A, which has a shorter incubation at 4 °C, we found spherical nanoparticles with a height of at least ~4 nm (Figure S1b). These data suggest that the spherical nanoparticles or nanogels aggregate into tubular structures when the incubation at low temperature increases. Whereas finding size-limited DNA structures at low concentrations was expected, having discrete multilayer thickness (Figure 3a) was surprising and may have been due to the out-of-plane flexibility of the sticky ends of the F-unit.

Large concentrations of DNA strands led to multilayer nanosheets with a large area coverage of at least 30 μm × 30 μm, which was the largest scan area characterized in our AFM investigation. ABC(20:20:200) using FAP-C led to nanosheets with large micrometre-sized pores and a thickness equivalent to up to three DNA duplex layers (Figure 3b; see additional AFM areas in Figure S2a, showing the sample could additionally form a network of connected nanotubes). ABC(20:20:200) using FAP-B showed similar nanosheets but with a thickness of a single DNA duplex layer (Figure S2b). As FAP-B had a shorter room temperature (RT) incubation time of 1 h, in contrast with the 1-day RT incubation time of FAP-C, we may assign the multilayer formation to a self-assembly process at RT in which F-units or middle-size structures stack to the surface of nanosheets via base pairing. At a much larger concentration of ABC(100:100:1000) using FAP-C, a heterogeneous sample was formed in which large coverage nanosheets with large particles on its surface and nanotubes could be observed (Figure S3).

At the intermediate concentrations of ABC(3,3,30), single-layer nanosheets with a large area coverage of at least 30 μm × 30 μm were formed at different annealing protocols. Whereas ABC(3,3,30) using FAP-B formed porous nanosheets (Figure 3c), the slower FAP-C formed a compact nanosheet whose minimal porosity could be resolved (Figure 3d and Figure S4a). Interestingly, the distance between pores in ABC(3,3,30) using FAP-C was 25 ± 1 nm (height profile in Figure 3d), which is in good agreement with the “diameter” of the designed nanopore as predicted by the oxDNA simulations (Section 3.2). To put this result into context, functional nanopores of such size or smaller can be fabricated using single three-dimensional DNA nanostructures [23,30,31], whereas our DNA nanosheets have the thickness resolution of a DNA duplex molecule, cover a large surface area, and have a high porosity density.

When characterizing ABC(3,3,30) nanosheets produced by slower annealing protocols, for example, SAP-A, we observed complete surface area coverage without large pores (Figure 3e and Figure S4b). Figure 3e shows two height profiles of a defect, in which we can observe the single-layer thickness of the nanopore and a small bump with the height of a second layer. Increasing the incubation time at RT by using SAP-B led to a heterogenous sample including folded-up nanosheets, scattered nanosheets, and particles (Figure S4c).

These results demonstrate that DNA nanosheets with a thickness of a single DNA duplex can be formed in solution and lie flat on a surface, i.e., are planar, as predicted by the oxDNA simulations (Section 3.2). Furthermore, the morphology of the nanosheets is governed by the concentration of the component strands and their annealing profile. This allows us to tune the morphology of our planar DNA nanosheets.

### 2.4. Self-Assembly at Solid/Liquid Interface

**Figure 4:**
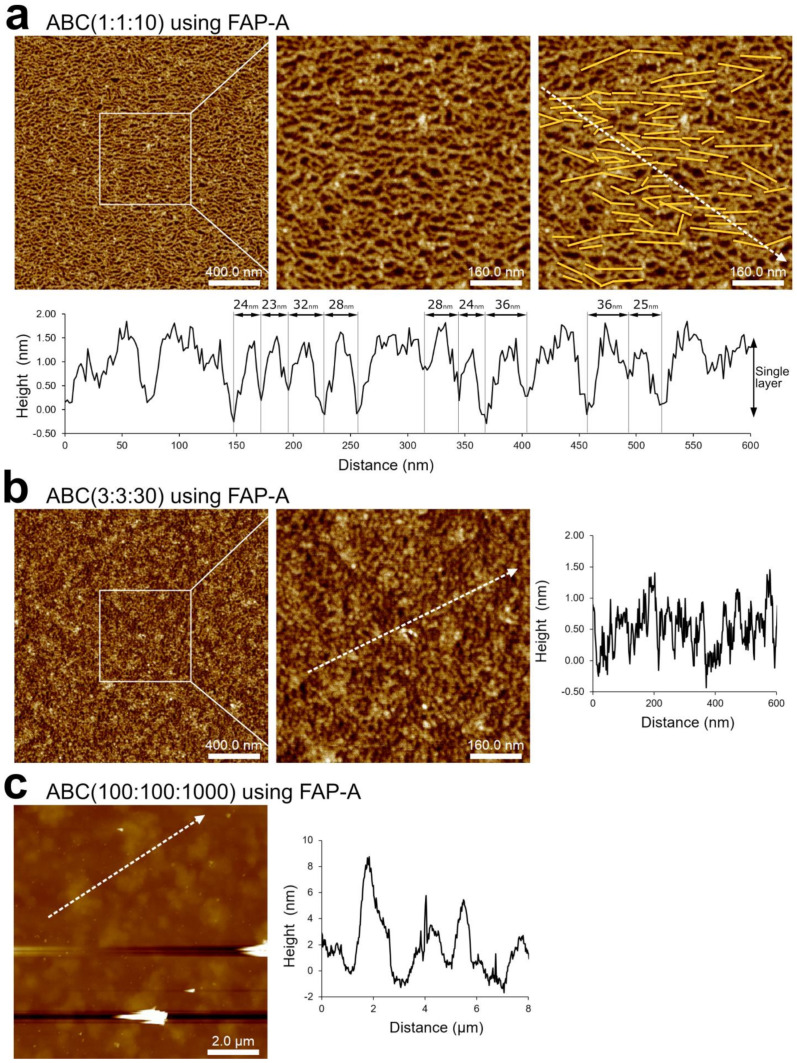
AFM characterization of DNA nanosheets self-assembled at the solid/liquid interface. (**a**) ABC(1:1:10) with FAP-A showing single-layer nanosheets with large area coverage. A zoomed-in image is shown (center). The same image with yellow lines (right) indicates the orientation of the quadrilateral pores, and a dashed line indicates the height profile shown at the bottom. [A] = 50 nM. (**b**) ABC(3:3:30) with FAP-A showing large area coverage with rough surface. [A] = 150 nM. Representative height profile is shown. (**c**) ABC(100:100:1000) with FAP-A showing large area coverage with a surface rougher than (**b**). [A] = 5 µM. Representative height profiles are shown.

Notably, some F-units in the liquid phase, deposited and incubated on a surface for AFM characterization, may be free in solution and bonding each other on the surface. Therefore, we opted to perform an interface-supported self-assembly [10,16,26,32] to understand this behavior and its effect on nanosheet morphology and porosity. In this regard, the same sample used for liquid phase self-assembly was prepared by introducing a piece of freshly cleaved mica substrate in the reaction chamber during annealing [32]. The mica substrate worked as a solid/liquid interface during the self-assembly (see Section 3). The results for the nanosheets assembled at the solid/liquid interface are shown in Figure 4 for the different concentrations of the component strands using FAP-A.

At the lowest concentration, i.e., ABC(1:1:10), AFM showed that nanosheets were formed with a quasi-random arrangement of F-units. Nevertheless, it was clear that connected F-units were oriented toward the horizontal axis of the image (see yellow lines in Figure 4a, top right) because of the asymmetry of the F-unit, and the thickness of the nanosheet agreed with the height of a single DNA duplex (Figure 4a, bottom). The distance between pores was 28 ± 5 nm (Figure 4a, bottom), which is still in the same order of magnitude as the “diameter” of the simulated nanopore (Figure 2f) and of a nanosheet assembled in the liquid phase (Figure 3d). Surface assembly produced nanopore sizes larger than in liquid phase, which led to more compact nanosheets (see, for instance, Figure 3e). The increase in pore size may have been due to the surface assembly imposing a 2D restriction with increased repulsion between DNA molecules. It is important to note that this finding rules out possible drying effects (during sample preparation, prior to AFM imaging) on the topology of the nanosheet: it is reasonable to assume that if drying effects were present, the nanopores would have been smaller and more compact [44]. However, it is still possible that the topology of DNA nanostructures much smaller than the nanosheets would be influenced by drying effects. The consistent height/thickness of the nanosheets and nanodiscs (from 1.5 nm to 2 nm and equivalent to the DNA duplex diameter) strongly indicates the programmable self-assembly of DNA rather than the random aggregation of single-stranded DNA components, which present much shorter heights than that of the DNA duplex [45] and coiled topologies [46].

At higher concentrations, ABC(3:3:30) and ABC(100:100:1000), AFM showed that the nanosheet becomes rougher, as shown in Figure 4b,c. These results strongly suggest that the superstructures obtained during liquid phase assembly are produced by the self-assembly in liquid rather than by the effect of the surface during sample deposition.

### 2.5. Self-Assembly at Liquid/Liquid Interface

Given that DNA nanosheets were successfully formed by the hierarchical assembly of DNA in the liquid phase and at solid interfaces, we next examined the presence of our F-unit and potential associated superstructures at an oil/aqueous interface. This interface offers a dynamic 2D space to study the mechanical properties of our system [1].

**Figure 5:**
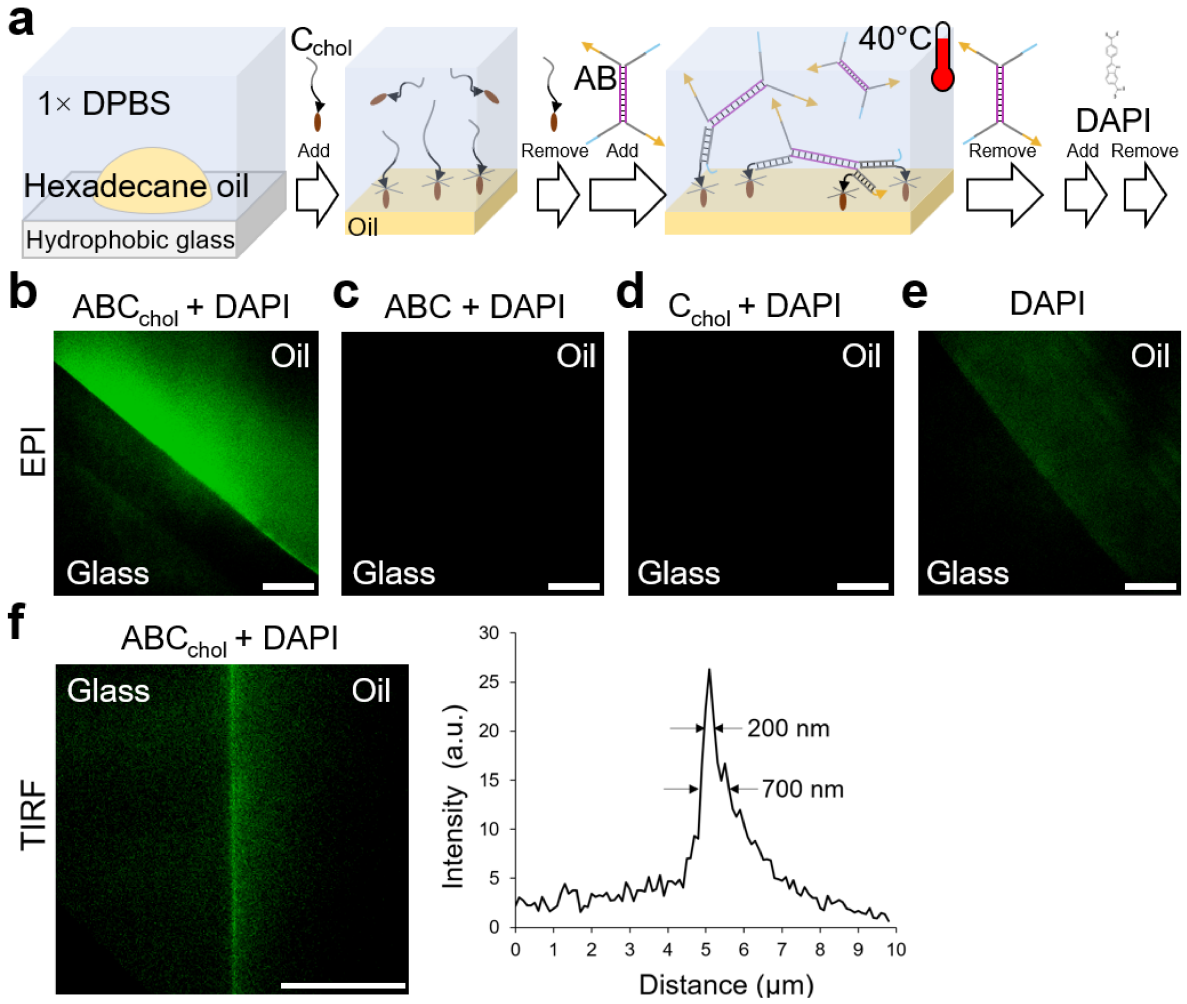
Fluorescence characterization of DNA self-assembled at the liquid/liquid interface. (**a**) Stepwise self-assembly. First, a droplet of hexadecane oil was deposited on a hydrophobic glass (triethoxy(octyl) silanated) immersed in 1× DPBS buffer. Second, C_chol_ was gently added to solution and rinsed after incubation. Third, a premixed solution of A and B was gently added, incubated at 40 °C, and immediately rinsed with 1× DPBS. (**b**–**e**) Epifluorescence microscopy of the F-units and the controls. The double-stranded DNA intercalator DAPI was used. The fluorescence of DAPI is shown as green. (**b**) Sample prepared with strands A, B, and C_chol_, as in (**a**). The fluorescence of DAPI was only observed on the oil region and not in the glass, indicating the presence of at least partially formed F-units. (**c**) Sample prepared with strands A, B, and C. The fluorescence of DAPI was not observed. (**d**) Sample prepared with strand C_chol_. The fluorescence of DAPI was not observed. (**e**) Sample was the oil alone without DNA. The fluorescence of DAPI was dimly observed on the oil region. (**f**) TIRF microscopy of sample in (**b**) showing the intersection of the glass and oil within the evanescence field. The intensity profile (right) for a region of interest demonstrates the presence of F-units at the oil/aqueous interface. Scale bars are 10 µm.

To confirm the placement of our F-unit at the oil/aqueous interface, we took a stepwise approach to induce assembly at the surface of hexadecane (see Section 3). In this approach, we initially anchored a cholesterol-modified strand C, called C_chol_, and later added a preannealed mixture of A and B (AB) incubated at 40 °C, which is above the melting temperature of the sticky ends but below the melting temperature of the arms and core (Figure 5a; melting temperatures are shown in Table S1); residual DNA strands were removed at each step after incubation, as described in the Section 3. We monitored the presence of F-units at the surface of the oil using fluorescence microscopy. We used DAPI, a DNA duplex intercalator, to allow imaging via fluorescence microscopy (DAPI was introduced into the system after sample preparation). When imaging the sample, we strategically imaged the edge of the oil droplet in contact with the glass to provide contrast and allow imaging within the focal depth of the microscopy and objective.

Figure 5b confirms the presence of F-units at the oil surface. A control using a C strand without cholesterol modification did not show fluorescence (Figure 5c), which demonstrated that AB did not attach to the oil surface via hybridization with C, which should not have been present due to the absence of cholesterol. In addition, this demonstrates that AB can only bind to the oil surface via hybridization with C_chol_, as expected. An additional control (Figure 5d), to verify the specificity of the DAPI dye to interact with DNA duplexes and not DNA strands, was performed using C_chol_ only. The control did not show fluorescence (Figure 5d), which further demonstrates that AB is required to make DAPI fluoresce due to intercalation, as shown in Figure 5b. An additional control (Figure 5e), to assess the interaction of DAPI with the oil surface, was performed. This control showed some fluorescence, indicating that DAPI could directly interact with the oil surface (Figure 5e); however, the level of fluorescence observed was low, and the presence of C_chol_ impeded the direct interaction of DAPI with the oil surface, as shown in Figure 5d.

Total internal reflection fluorescence (TIRF) microscopy, a technique that allows characterizing fluorescence emitted within a ~100 nm evanescence region standing from a thin glass coverslip surface [8,13,14,28], was used to monitor the presence of F-units and potential associated superstructures at the oil/aqueous interface (Figure 5f). The results of TIRF microscopy confirmed that AB duplexes were anchored to C_chol_ (at the oil/aqueous interface), but it did not inform us whether a superstructure with a single-molecule DNA thickness was present, as instead observed in Section 3.3 and Section 3.4. However, we could assume that due to spatially separated C_chol_ strands at the oil/aqueous interface (see Text S2 for the rationale to calculate a ~6 nm separation distance), AB duplexes formed complete F-units by having their four arms anchored (hybridized) to C_chol_, whereas the rest of anchored AB duplexes could have their arms and unhybridized sticky ends protruding toward the solution (partially formed F-units). The unhybridized sticky ends promoted the binding of AB duplexes diffusing in solution (during AB duplex incubation), which would further promote the formation of a superstructure whose DNA density decreases further from the oil/aqueous interface. Hence, our motivation to incubate the sample with AB duplexes at 40 °C, as described above, was to minimize the formation of thicker superstructures. This incubation temperature is above the melting temperature of the F-unit’s sticking ends and below the melting temperature of the core (AB duplex), and sufficiently allows AB duplexes to anchor to C_chol_ at the oil/aqueous interface. The fact that the excess of AB duplexes was immediately removed could lead, in the ideal case, to a single layer of F-units and AB duplexes; however, in the practical case, the remaining AB duplexes increase the thickness of the superstructure. Once at room temperature, the sticky ends could hybridize with their complementary strands, if available, further enhancing the connectivity of the assembly forming a DNA nanosheet superstructure. Indeed, the fluorescent region observed in Figure 5f could have been due to this superstructure.

#### Interfacial Mechanical Properties

The AFM evidence of nanosheet formation from aqueous solutions (Figure 3 and Figure 4), together with the demonstration by fluorescence of the presence of F-units at the oil/aqueous interface, allowed us to reasonably assume the formation of DNA nanosheets also at the oil/aqueous interface. To further investigate this system, the mechanical properties of DNA nanosheets assembled in solution and at the oil/aqueous interface were characterized via interfacial shear rheology. This technique, carried out by shearing a probe such a Du Nouy ring or bioconical discs at a liquid/liquid interface [44,45], is better suited than dilatational rheology (e.g., based on pendant droplets) as it is not sensitive to surface tension and allows directly probing the mechanics of the associated interfacial assemblies. In addition, interfacial shear rheology was found to be significantly more sensitive to the shear mechanics of liquid/liquid interfaces than atomic force probe microscopy owing to the complexity of the parameters (e.g., surface tension, electrostatic forces, and precise probe contact area) impacting the associated displacement profiles [46].

**Figure 6:**
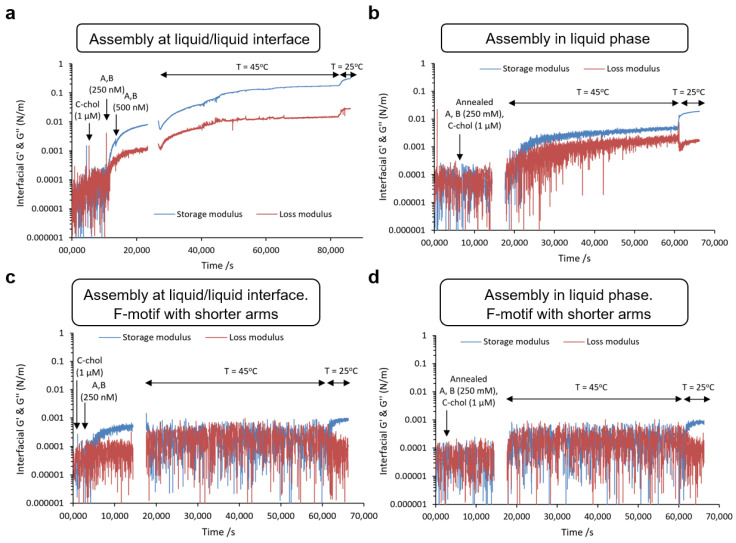
Characterization of the interfacial mechanical properties of the DNA nanosheet superstructure. (**a**–**d**) After equilibration, samples were heated to 45 °C and then cooled to 25 °C. (**a**) DNA nanosheet was prepared at the oil/aqueous interface, as in Figure 4. (**b**) DNA nanosheet was prepared in liquid and deposited on the oil surface. (**c**) DNA nanosheet was prepared at the oil/aqueous interface, as in Figure 5, but with shorter DNA arms (melting temperature below 45 °C). (**d**) DNA nanosheet was prepared in solution and deposited on the oil surface but with shorter DNA arms.

The addition of C_chol_ had a negligible effect on interfacial shear rheology (beyond the detection limit of our instrument, considering the non-negligible drag associated with both liquid phases), in agreement with the expected surface-bound diffusion of non-crosslinked surfactants at liquid interfaces (Figure 6a,c) [1,37]. Upon further addition of AB at 250 nM, both interfacial storage and loss shear moduli increased, with a sharper rise in the storage component (Figure 6a). Following the addition of another load of AB (to a final concentration of 500 nM), the interfacial storage shear modulus rose to 10 mN/m, a level comparable to that observed for globular protein assemblies [1,35]. Following from this assembly, the temperature of the system was raised to 45 °C, above the melting temperature of the sticky ends, triggering a notable further rise in the interfacial shear moduli, with the storage component reaching >100 mN/m, consistent with the bridging of F-units and the formation of a crosslinked network of macromolecules at the liquid/liquid interface. Finally, after allowing a modulus plateau to establish, the temperature of the system was reduced back to 25 °C. This resulted in the annealing and strengthening of the network, with a further rise in interfacial storage shear modulus to 300 mN/m (Figure 6a).

In contrast, the assembly of AB with shorter sticky ends initially resulted in a modest increase in the interfacial shear storage modulus at low temperature but did not result in a significant further increase in the interfacial storage modulus at 45 °C, the temperature at which the shorter sticky ends were expected to disassemble (Figure 6c). In fact, the interfacial shear loss modulus caught up with the storage component, indicating the formation of a viscous fluid interface lacking extensive crosslinked network characteristics. The interfacial storage shear modulus increased back to 1 mN/m upon cooling the system to 25 °C. In addition, the assembly of annealed ABC_chol_ formed in the liquid phase led to > 1 order of magnitude lower interfacial shear moduli both below and above the melting temperature (Figure 6b). When short arms were used, interfacial shear mechanics was observed comparable to that observed with assembly directly at the interface (Figure 6d). Overall, the data indicate that the interfacial assembly of the DNA nanosheet results in the formation of a crosslinked macroscopic network associated with a significant increase in interfacial shear moduli and elasticity.

The initial lack of increase in interfacial shear moduli (prior to the introduction of AB) and the limited level of crosslinking observed with short-arm F-units are in good agreement with interfacial shear mechanical properties of non-crosslinked macromolecules and protein assemblies that display interfacial shear storage moduli in the range of 1–30 mN/m and limited interfacial elasticity [1,37,47]. In contrast to dilatational shear moduli, which are sensitive to changes in surface tension associated with molecular assembly at the liquid/liquid interface, interfacial shear moduli are far less sensitive to such processes in the absence of further crosslinking. Macromolecules and small protein aggregates retain the ability to diffuse at corresponding interfaces; therefore, they do not contribute to interfacial elasticity and do not substantially raise the interfacial shear moduli (below contributions from sub/upper phase viscous drags). In contrast, upon assembly of full-length F-units, the interfacial shear storage modulus was raised to 100 mM/m and further increased upon annealing, with a gap with the loss component of >1 order of magnitude. Therefore, our results are consistent with the designed F-unit sustaining the formation of a dense crosslinked network of macromolecules, being able to significantly contribute to interfacial mechanical properties and elasticity.

Interestingly, extrapolating the interfacial shear storage moduli measured after the annealing of DNA nanosheets to bulk moduli indicated Young’s moduli near 450 MPa, comparable to those previously measured for poly(L-lysine) nanosheets, the most rigid molecular interfacial assembly reported to date (with an interfacial shear storage modulus near 2 N/m, but a thickness of 6–10 nm, corresponding to Youngs’ moduli in the range of 600–1000 MPa) [36]. However, unlike the highly disordered characteristic of PLL nanosheets [37], DNA nanosheets have well-defined architecture and porosity.

Based on the morphology of the nanosheets that can be observed in Figure 4a, the DNA nanosheets that formed at liquid/liquid interfaces could be predicted to display porosities above 40%. As a result, the Young’s modulus associated with F-units could be estimated to be at least 750 MPa, potentially near 1 GPa. This corresponds to strikingly high moduli for DNA structures, increasing the possibility of applying DNA nanosheets to the structuring of soft matter.

Although significantly higher than the moduli typically reported for DNA hydrogels (1 Pa to a few tens of kPa) [48,49], these values are consistent with the low molecular density typically associated with such gels. In comparison, dense DNA assemblies at the surface of cantilevers were reported to display compressive moduli in the range of a few tens of MPa to 100 MPa [50], although associated densities remain low compared with those achieved in the present nanosheets. Nanosheets mechanics is, however, in excellent agreement with the stretching moduli measured for double-stranded DNA, in the range of 1000 pN per molecule, corresponding to GPa Young’s moduli for dense perfectly aligned DNA-based materials [51].

## 3. Materials and Methods

### 3.1. F-Unit Design and DNA Sequences

DNA sequences were initially generated using an existing approach for sequence generation [52,53,54] and then sequences were manually tuned. Each tuning was simulated in Nucleic Acid Package (NUPACK) [55] to reach high concentrations of target structures and in Oligoanalizer to prevent unintended secondary structures and self-dimerizations. The optimized sequences, strands A, B, and C, are shown in Text S1. The length of each DNA duplex was rationally selected to achieve hierarchical self-assembly based on their melting temperature under a temperature annealing profile (see Table S1 for calculated melting temperatures). All oligonucleotides and modifications were purchased from integrated DNA technologies (IDT). The different samples are denoted by ABC(m:n:p), where [A]:[B]:[C] = m:n:p, and m = n = p/10.

### 3.2. oxDNA Simulations

Simulations were performed using the most recent implementation of the oxDNA model [56] in LAMMPS [57]. Briefly, this is a single nucleotide resolution model of DNA that has been thoroughly characterized and used in a broad range of systems in biology, biophysics, and nanotechnology [41]. In the oxDNA model, the interactions between different nucleotides are set through potentials accurately representing the hydrogen bonding between complementary bases, the connectivity of the sugar-phosphate backbone, and the excluded volume between nucleotides, including the stacking, coaxial-stacking, and cross-stacking forces. The initial configuration of an F-unit (without the cholesterol part) was built using oxView [58]. Simulations were evolved with implicit solvent (Langevin dynamics) at RT and [NaCl] = 0.15 M.

### 3.3. Liquid-Phase Assembly of DNA Nanosheets and AFM Characterization

The DNA mixture was introduced into a microtube reaction chamber for annealing using a PCR machine (Hybrid Sprint PCR Thermal Cycler, Thermo Scientific) with the following different annealing temperature profiles: Slow annealing protocol A (SAP-A): 5 min at 90 °C, −1 °C/5 min, 3 h at 68 °C, −1 °C/5 min, 3 h at 59 °C, −1 °C/5 min, 6 h at 42 °C, −1 °C/5 min, 6 h at 40 °C, and hold at 4 °C for 2 min. Slow annealing protocol B (SAP-B): 5 min at 90 °C, −1 °C/5 min, 3 h at 68 °C, −1 °C/5 min, 3 h at 59 °C, −1 °C/5 min, 6 h at 42 °C, −1 °C/5 min, 6 h at 40 °C, and hold at 4 °C for at least an hour. Fast annealing protocol A (FAP-A): 5 min at 90 °C, 10 min at 68 °C, 10 min at 59 °C, 1 h at 42 °C, −1 °C/5 min, and hold at 25 °C for 30 min. Fast annealing protocol B (FAP-B): 5 min at 90 °C, 10 min at 68 °C, 10 min at 59 °C, 1 h at 42 °C, −1 °C/5 min, and hold at 25 °C for 1 h. Fast annealing protocol C (FAP-C): 5 min at 90 °C, 10 min at 68 °C, 10 min at 59 °C, 1 h at 42 °C, −1 °C/5 min, and hold at 25 °C for 1 day. The selection of these temperatures was consistent with the melting temperatures of the duplex components of the F-unit (see Table S1). Buffer solution was 1× TAE (ThermoFisher), which was supplemented with 12.5 mM MgCl_2_ (Fisher Scientific) in Milli-Q water (mQ).

The characterization of the topography of the formed DNA nanosheets was performed by AFM. Because DNA superstructures strongly bind onto mica substrates due to the presence of Mg^+2^ divalent cations in solution (and weaker for Na^+1^ monovalent cations), the characterization of nanosheets via AFM was straightforward. The samples were prepared as follows: First, 5 µL of annealed sample was deposited on freshly cleaved mica for 1 h in a moist chamber. Then, the mica was immersed for 3 s in mQ water, gently stirring it. The surface of mica with was gently blow-dried with inert gas. The sample was left to dry, and, in the meantime, the AFM system was prepared. AFM in air was performed using a Bruker dimension icon AFM in tapping mode with ScanAsyst air tips (tip radius 12 nm). Images were processed in Bruker Nanoscope Analysis software (v1.7) and Gwyddion software (v2.55) [59].

### 3.4. Solid/Liquid Interface Assembly of DNA Nanosheets and AFM Characterization

A piece of freshly cleaved mica was introduced into the microtube reaction chamber containing the DNA mixture for annealing. Then, the mica was removed and immersed for 3 s in mQ water, with gently stirring. The surface of mica with was gently blow-dried with inert gas. The sample was left to dry; in the meantime, the air AFM system was prepared. AFM was performed using the same procedure as for the liquid-phase assembled nanosheets.

### 3.5. Liquid/Liquid Interface Assembly of DNA Nanosheets and Fluorescence Microscopy Characterization

A coverslip glass (Agar Scientific) was functionalized with triethoxy(octyl)silane in toluene (Sigma-Aldrich) to enhance its hydrophobicity. A reaction chamber on top of the resulting hydrophobic glass coverslip was prepared. A cloning cylinder was glued (ethyl 2-cyano acrylate; loctite) onto the surface of the hydrophobic glass. The chamber was filled with 150 µL of 1× DPBS buffer (Dulbecco’s phosphate-buffered saline, ThermoFisher). Then, 5 µL hexadecane oil droplet was introduced through the 1× DPBS solution and gently deposited on the functionalized surface using a pipette. The drop remained attached to the surface and spread due to hydrophobic interactions. Next, 1 µL of C_chol_ (which is the C strand with a cholesterol modification attached to its 3′ end via a triethylene glycol (TEG) linker. See Text S1 for sequences) was added to the reaction chamber, to a final 100 nM concentration, gently stirred with the pipette, and then incubated for 30 min. The solution in the chamber was carefully exchanged with new 1× DPBS to remove excess C_chol_ in such a way that the same 100 µL total volume in the chamber remained. A mixture of A and B strands, AB(1:1), was hybridized at RT for 1 h in parallel. 1 µL of AB(1:1) was added to the reaction chamber to a final concentration of 500 nM. The chamber was heated in a hot plate at 40 °C for 10 min, and the solution was immediately exchanged with 1× DPBS by adding and removing equal amounts, e.g., 50 µL of 1× DPBS. The chamber was incubated for 30 min. A further solution exchange with 1× DPBS was performed. Control samples were prepared using the same protocol but with relevant oligonucleotide sequences.

To perform fluorescence microscopy, the double-stranded DNA dye intercalator DAPI was added to the chamber and incubated. Then, a solution exchange with 1× DPBS was carried out. Experiments were performed using SM710 ELYRA PS.1 in epifluorescence (EPI) mode or in total internal reflection fluorescence (TIRF) mode with a 405 nm laser excitation and using a Plan-Apochromat 100×/1.46 oil objective. The sample was observed with EPI and TIRF microscopy to gain insight into the DNA nanosheet superstructure 3D coverage on the surface of the hexadecane oil and to confirm that the DNA nanosheet was placed on the surface of the hexadecane oil, respectively.

### 3.6. Interface Mechanical Characterization

Interfacial shear rheology was carried out using a Discovery Hydrid Rheometer (DHR-3) from TA Instruments, fitted with a Du Nouy Ring geometry and a Delrin trough with a circular channel. The Du Nouy ring (platinum–iridium wire) had a diamond-shaped cross-section of 1000 mm and a radius of 10 mm. To set up the ring at liquid/liquid interfaces, 19 mL of 1× DPBS was introduced in the trough, and the ring was lowered to the interface using axial force monitoring (micron resolution). Upon contact, the ring was lowered by a further 500 mm prior to introducing the upper liquid phase (hexadecane, Sigma-Aldrich; 18 mL). Time sweeps were performed at a frequency of 0.1 Hz and temperature of 25 °C, with a displacement of 1.0 × 10^−3^ rad (strain of 1%), to follow the response of the DNA nanosheets at the corresponding interfaces. Associated baselines were typically in the range of 10^−5^–10^−4^ N/m. Frequency sweeps were carried out with displacements of 1.0 × 10^−3^ rad and amplitude sweeps at a frequency of 0.1 Hz. Following equilibration of the naked liquid/liquid interface (DPBS-hexadecane), DNA strands were introduced into the aqueous subphase of the system using a thin canular. The trough was heated while monitoring the temperature in the aqueous phase using a Peltier controlled by a thin thermocouple (equilibrated in the range of 45–48 °C). This temperature range was selected to be above the *T_m_* of the arms of the F-unit. To allow assembly of the arms, the system was cooled to 25 °C. To enable stepwise nanosheet formation, C_chol_ was added to the aqueous phase to a final 1 μM concentration and allowed to incubate for 20 min. Then, a mixture of AB(1:1) was added to the aqueous phase to a final concentration of 250 nM. After each DNA strand addition, the system was gently stirred. The system was incubated for 3 h at RT before oscillatory rheology. For liquid-phase nanosheet formation (preassembly prior to injection), ABC_chol_(1:1:4) was mixed in a total volume of 500 μL of 1× DPBS and annealed using FAP-B. The mixture of ABC_chol_(1:1:4) was added to the aqueous phase to a final concentration of 1μM C_chol_. The system was incubated for 3 h at RT before oscillatory rheology.

## 4. Conclusions

We demonstrated, as confirmed by AFM, the formation of DNA nanosheets with the discrete thickness resolution of a single-molecule DNA duplex via a programmed and hierarchical self-assembly approach, in which a minimal set of three DNA strands assembled into F-units, and then the F-units into nanosheets. This design enabled us to directly control the structural, thermodynamical, and (bio)functionalization aspects of the DNA F-unit, and, hence, control the properties of the nanosheets. Nanosheets were formed in the liquid phase, at the solid/liquid interface, and at the liquid/liquid interface. Our study workflow included design, simulation validation, and characterization. To achieve 2D nanosheets, planarity was the critical aspect of the rationale in the design of the F-units, and the planarity of the F-unit was verified by oxDNA simulations. In addition, our simulations provided insight into the quadrilateral geometry of the nanosheet’s nanopores and the predicted nanopore’s planarity and size. These predictions were confirmed by characterizing the nanosheets with AFM. AFM revealed nanodiscs and nanosheets assembled in the liquid phase with thicknesses equivalent to single and multiple DNA duplexes. Nanosheets with single DNA duplex thickness and a large surface area coverage of at least 30 μm × 30 μm were assembled in the liquid phase and at the solid/liquid interface. Depending on the self-assembly conditions, the diameter of the nanosheet’s nanopore varied between 25 ± 1 nm and 28 ± 5 nm, in agreement with predictions from our oxDNA simulations. Finally, nanosheet superstructures were successfully assembled at the liquid/liquid interface, as confirmed by fluorescence microscopy. Interfacial shear rheology also demonstrated that the resulting nanosheets displayed strong elastic mechanical properties, depending on the design of the F-units and their assembly conditions. These properties were also strikingly dependent on the temperature of assembly and annealing of the system, further confirming that the F-unit design controlled the formation of crosslinked DNA networks with mechanical properties consistent with the formation of dense mechanical interlocked DNA structures.

This study demonstrates, as a proof of concept, the formation of a DNA-based macroscopic system achieving long-range mechanical function. The rational design of DNA technologies paves the way toward the tailoring of liquid/liquid interfaces and systems displaying controllable mechanical and potential responsive properties. The nanosheets presented here could be used in different applications ranging from biotechnology to materials science. For example, DNA nanosheets could replace protein- and polymer-based nanosheets that were recently demonstrated to enable cell adhesion at liquid interfaces for the scale-up of cell manufacture [38,39]. Another field of application may also be the “sculpting” of liquid/liquid interfaces, recently proposed to allow the control of microfluidic systems using 3D-printing extrusion platforms [33]. The combination of experimental and computational work [8] offers powerful opportunities to guide and validate the design of functional structures and large superstructures fabricated via DNA self-assembly; in this context, the workflow and study provided here will be useful to inspire the design and use of advanced and functional nanosheet superstructures.

## Data Availability

The data presented in this study are contained in the Main Text and Supplementary Materials.

## Supplementary Materials

The following supporting information can be downloaded at https://www.mdpi.com/article/10.3390/molecules28093686/s1, Text S1—Table S1: The DNA sequences for F-unit; Figure S1: AFM characterization of F-unit superstructures assembled in liquid phase. (a) ABC(1:1:10) using FAP-C. A height profile is shown. [A] = 50 nM. (b) ABC(1:1:10) using SAP-B. The height profile of encircled particles and asymmetrical structures are shown. [A] = 50 nM. (c) ABC(0.6:0.6:6) using SAP-A. Three scanning areas (top), and the height profile of particles (bottom) are shown. [A] = 30 nM; Figure S2: AFM characterization of F-unit superstructures assembled in liquid phase (a) ABC(20:20:200) using FAP-C. Two scanning areas are shown. Area 1 shows a multilayer nanosheet. Area 2 shows a network of connected nanotubes. [A] = 1 μM. (b) ABC(20:20:200) using FAP-B. A height profile is shown. [A] = 1 μM; Figure S3: AFM characterization of F-unit superstructures assembled in liquid phase. ABC(100:100:1000) using FAP-C. Four scanning areas are shown. Area 1 shows a large coverage of a single-layer nanosheet with particles on top. [A] = 5 μM; Figure S4: AFM characterization of F-unit superstructures assembled in liquid phase. (a) ABC(3:3:30) using FAP-C. Two scanning areas, and a representative height profile are shown. [A] = 150 nM. (b) ABC(3,3,30) using SAP-A. Two scanning areas are shown. [A] = 150 nM. (c) ABC(3,3,30) using SAP-B. Three scanning areas (top) with representative height profiles (bottom) are shown. [A] = 150 nM; Text S2. The two references from the Supplementary Materials are included as references [60,61] in the Main Text.

## Sample Availability Statement

Samples of the compounds are not available from the authors.
