# Supplementary material for: Programmed Self-Assembly of DNA Nanosheets with Discrete Single-Molecule Thickness and Interfacial Mechanics: Design, Simulation, and Characterization
Source: Molecules. 2023 Apr 24;28(9):3686. doi: 10.3390/molecules28093686 (PMC10180480; doi:10.3390/molecules28093686)
Supplement: Supplementary file 1 [file molecules-28-03686-s001.zip › molecules-2291550-supplementary.pdf]

Supplementary Materials for:

Programmed Self-Assembly of DNA Nanosheets with Discrete Single-Molecule Thickness and Interfacial Mechanics: Design, Simulation, and Characterization

Keitel Cervantes-Salguero [1,*,†], Yair Augusto Gutiérrez Fosado [2], William Megone [3], Julien E. Gautrot [3,*] and Matteo Palma [1,*]

1 Department of Chemistry, School of Physical and Chemical Sciences, Queen Mary University of London, London E1 4NS, UK
2 School of Physics and Astronomy, University of Edinburgh, Peter Guthrie Tait Road, Edinburgh EH9 3FD, UK
3 School of Engineering and Materials Science, Queen Mary University of London, Mile End Road, London E1 4NS, UK
* Correspondence: keitelcervantes@gmail.com (K.C.-S.); j.gautrot@qmul.ac.uk (J.E.G.); m.palma@qmul.ac.uk (M.P.)
† Current address: Micron School of Materials Science and Engineering, Boise State University, Boise, ID 83725, USA.

Text S1

The DNA sequences for F-unit:

A: GTGCTGTATC CCATGTTACCTTCCAC CGACTGACTCTGACTGCGATG CCATGTTACCTTCCAC CTGATCTCTG
B: GATACAGCAC CCATGTTACCTTCCAC CATCGCAGTCAGAGTCAGTCG CCATGTTACCTTCCAC CAGAGATCAG
C_{chol}: GTGGAAGGTAACATGG AAAAA - TEG - cholesterol

The DNA sequences for F-unit with short arms:

A_{short}: GTGCTGTATC TACCTTCCAC CGACTGACTCTGACTGCGATG TACCTTCCAC CTGATCTCTG
B_{short}: GATACAGCAC TACCTTCCAC CATCGCAGTCAGAGTCAGTCG TACCTTCCAC CAGAGATCAG
C_{chol}: GTGGAAGGTAACATGG AAAAA - TEG - cholesterol

Table S1: The DNA sequences for F-unit

			**Melting temperature (T_m)**	
	**Part**	**Sequence**	**DPBS***	**$MgCl_2$****
**F-unit**	Sticky ends	SE1: GTGCTGTATC<br>SE1*: GATACAGCAC	31.2 ºC (0.1 µM)<br>37 ºC (1 µM)	35.7 ºC (0.1 µM)<br>41.7 ºC (1 µM)
		SE2: CTGATCTCTG<br>SE2*: CAGAGATCAG	29 ºC (0.1 µM)<br>34.9 ºC (1 µM)	33.4 ºC (0.1 µM)<br>39.6 ºC (1 µM)
	Arm	CCATGTTACCTTCCAC	52.2 ºC (0.1 µM)<br>56.3 ºC (1 µM)	54.9 ºC (0.1 µM)<br>59.1 ºC (1 µM)
	Core	CGACTGACTCTGACTGCGATG	63.7 ºC (0.1 µM)<br>66.9 ºC (1 µM)	65.2 ºC (0.1 µM)<br>68.3 ºC (1 µM)
**F-unit (short arm)**	Arm	TACCTTCCAC	31.4 ºC (0.1 µM)<br>37.6 ºC (1 µM)	35.9 ºC (0.1 µM)<br>42.3 ºC (1 µM)

Oligoanalizer simulation was performed with the following conditions.

* 1x DPBS buffer: 150 mM Na^+
** 1x TAE/12.5 mM $MgCl_2$ buffer: 50 mM Na^+ and 12.5 mM Mg^{+2}

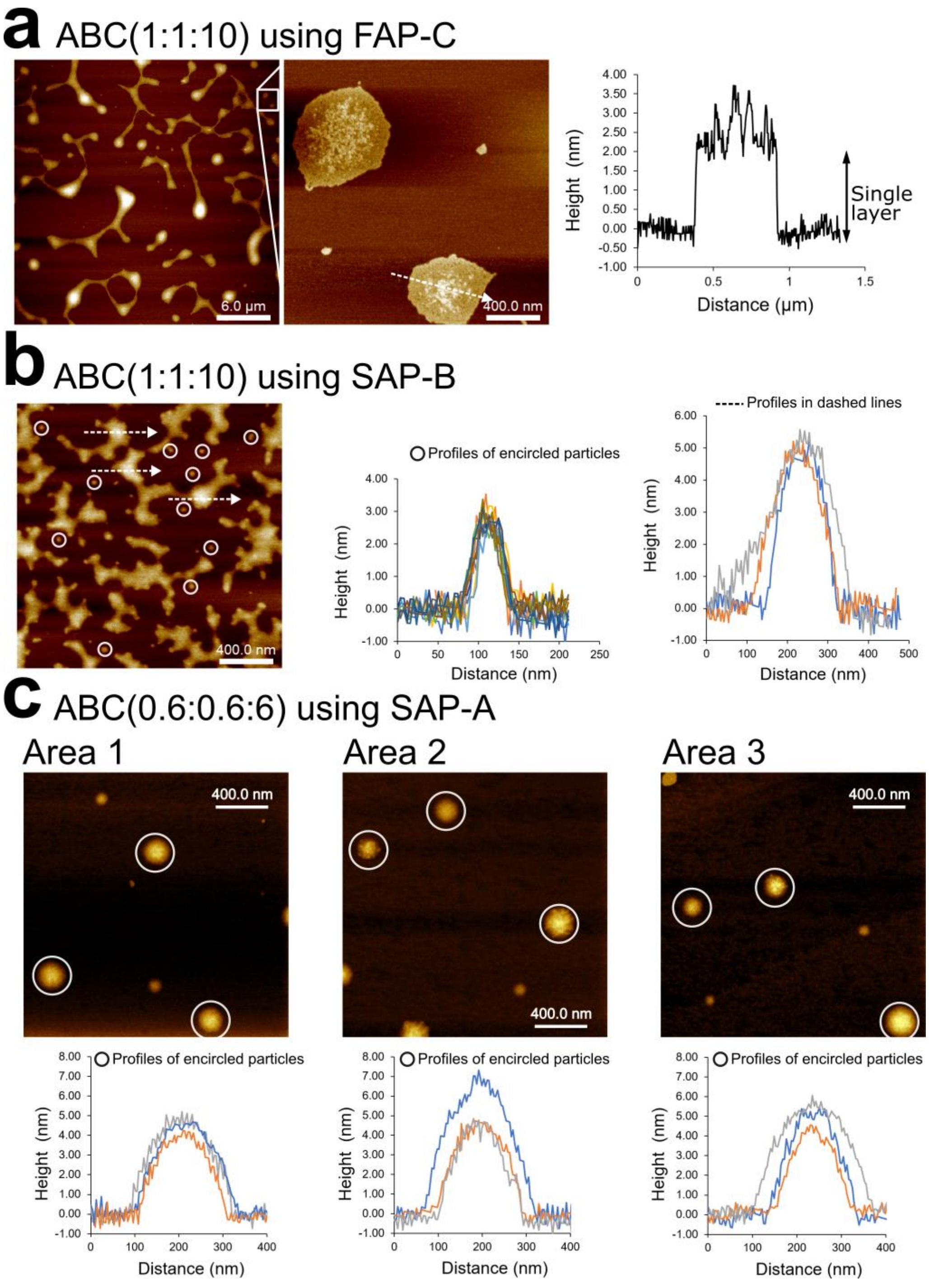

Figure S1. AFM characterization of F-unit superstructures assembled in liquid phase. (**a**) ABC(1:1:10) using FAP-C. A height profile is shown. [A] = 50 nM. (**b**) ABC(1:1:10) using SAP-B. The height profile of encircled particles and asymmetrical structures are shown. [A] = 50 nM. (**c**) ABC(0.6:0.6:6) using SAP-A. Three scanning areas (top), and the height profile of particles (bottom) are shown. [A] = 30 nM.

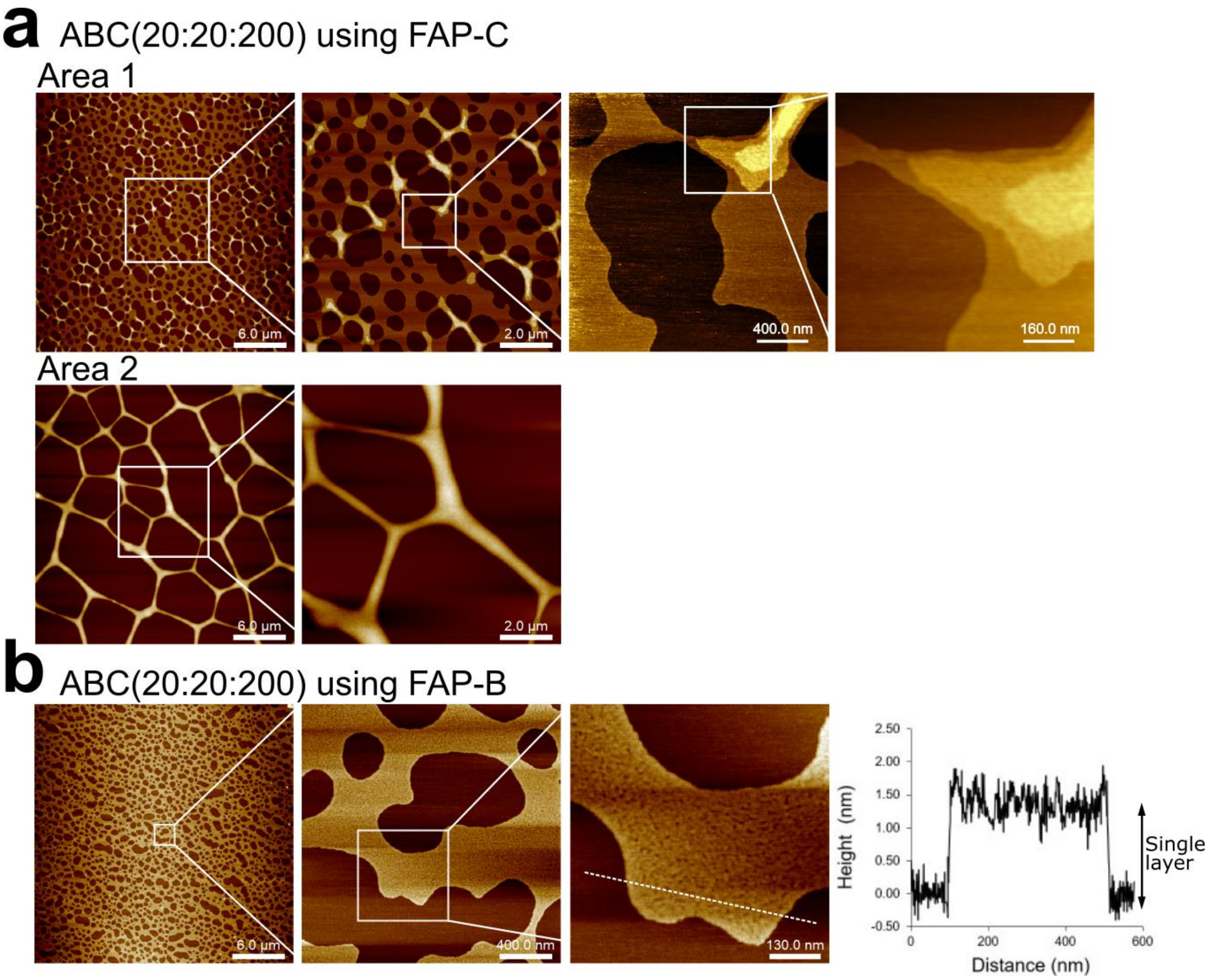

Figure S2. AFM characterization of F-unit superstructures assembled in liquid phase (**a**) ABC(20:20:200) using FAP-C. Two scanning areas are shown. Area 1 shows a multilayer nanosheet. Area 2 shows a network of connected nanotubes. [A] = 1 µM. (**b**) ABC(20:20:200) using FAP-B. A height profile is shown. [A] = 1 µM.

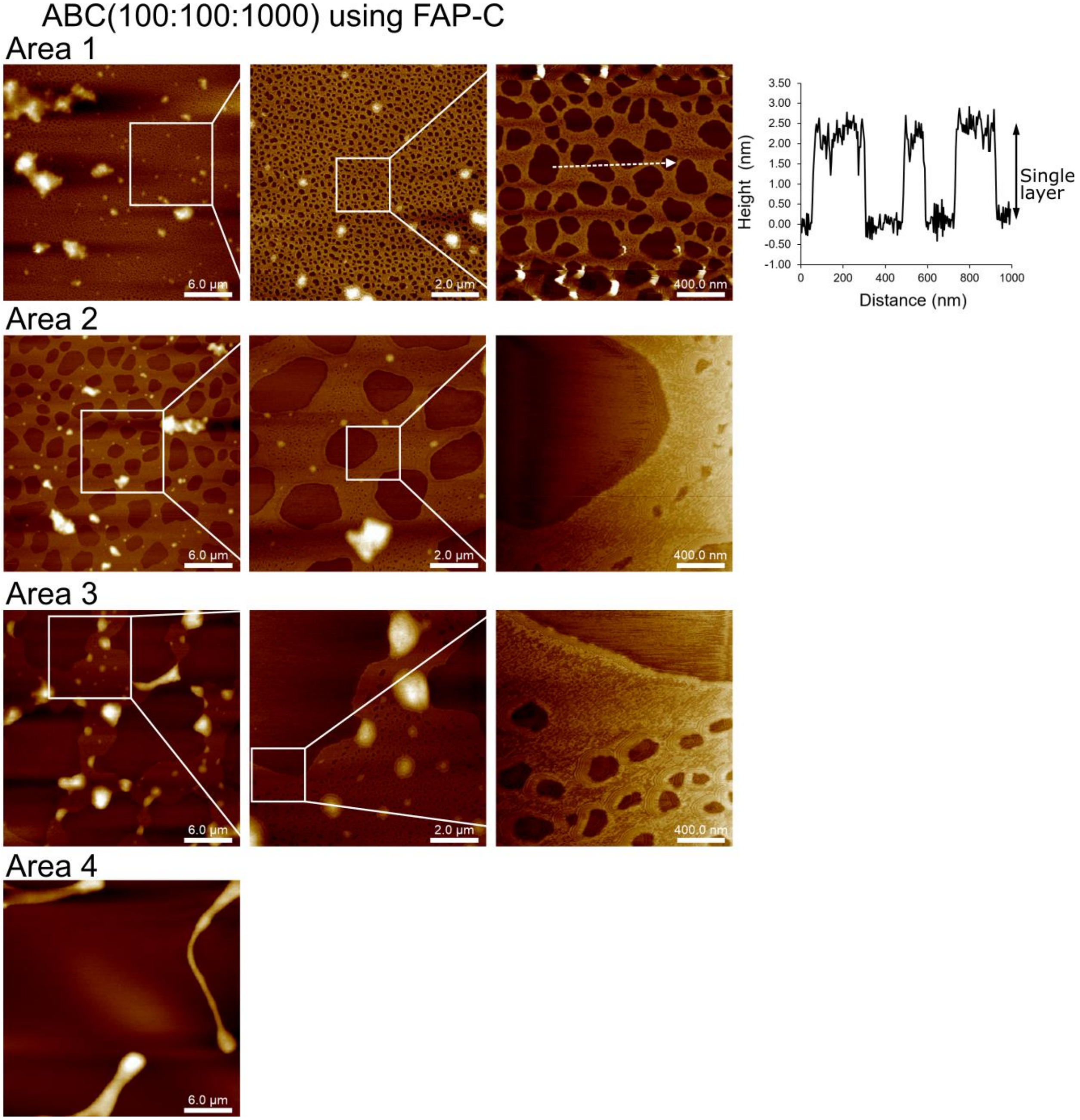

Figure S3. AFM characterization of F-unit superstructures assembled in liquid phase. ABC(100:100:1000) using FAP-C. Four scanning areas are shown. Area 1 shows a large coverage of a single-layer nanosheet with particles on top. [A] = 5 µM.

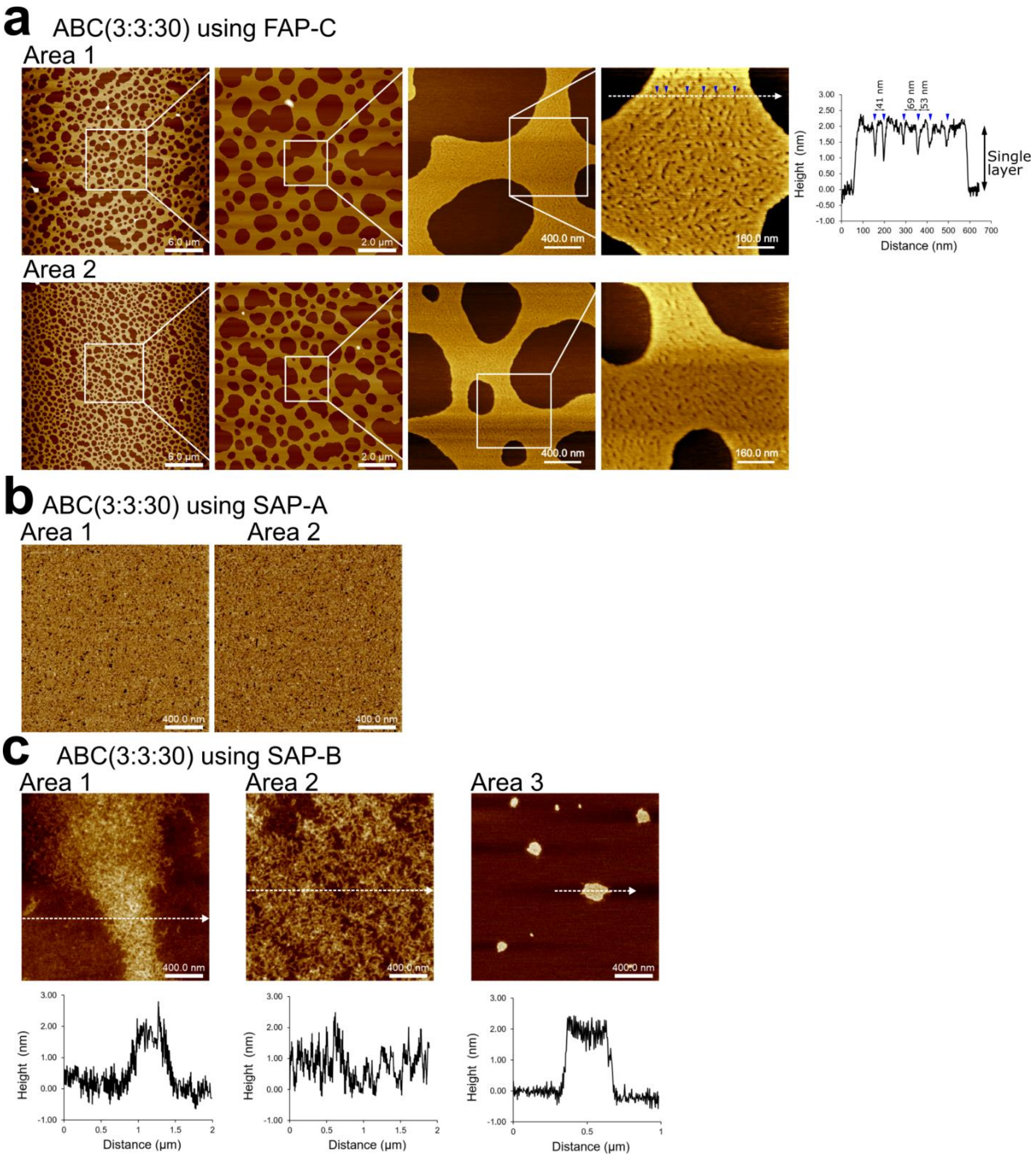

Figure S4. AFM characterization of F-unit superstructures assembled in liquid phase. (**a**) ABC(3:3:30) using FAP-C. Two scanning areas, and a representative height profile are shown. [A] = 150 nM. (**b**) ABC(3,3,30) using SAP-A. Two scanning areas are shown. [A] = 150 nM. (**c**) ABC(3,3,30) using SAP-B. Three scanning areas (top) with representative height profiles (bottom) are shown. [A] = 150 nM.

Text S2

By assuming that the surface density of C_{chol} (21 bases plus the hydrophilic flexible TEG linker) at the oil/aqueous interface is limited by the radius of gyration of DNA and the electrostatic repulsion between C_{chol} strands, the space occupied by each C_{chol} can be approximated as the sum of the radius of gyration of the DNA strand and the length of the flexible TEG linker, which are ~2 nm (interpolating from [1]) and ~2.25 nm, [2] respectively. As a result, the cholesterol of C_{chol} could be separated ~6 nm, which could be longer due to the electrostatic repulsion between the DNA's backbones. This separation is in the same order of magnitude as the distance between cholesterol modifications in the F-unit design in Section 3.1 of the Main Text, i.e., a minimum distance of ~3 nm and a maximum distance of ~14 nm.

Supplementary Information references

1. Sim, A.Y.L.; Lipfert, J.; Herschlag, D.; Doniach, S. Salt Dependence of the Radius of Gyration and Flexibility of Single-Stranded DNA in Solution Probed by Small-Angle X-ray Scattering. *Phys Rev E* **2012**, *86*, 21901, doi:10.1103/PhysRevE.86.021901.

2. Palma, E.; Klapper, D.G.; Cho, M.J. Antibodies as Drug Carriers III: Design of Oligonucleotides with Enhanced Binding Affinity for Immunoglobulin G. *Pharm Res* **2005**, *22*, 122–127, doi:10.1007/s11095-004-9017-z.
